# Innovative approach to recurrent giant cell tumor of the hand: free osteoarticular metatarsal transfer with simplified donor site management

**DOI:** 10.1093/jscr/rjaf032

**Published:** 2025-02-28

**Authors:** Ibad Sha I, Arun Kumar Seshadrinath

**Affiliations:** Lifeline Multi-speciality Hospital, No. 46, 14th Mile, Kayamkulam—Pathanapuram Road, Adoor 691554, India; Department of Orthopaedics, Sri Balaji Medical College, Lawyer Jaganathan St, Ramapuram, Guindy, Chennai 600032, India

**Keywords:** giant cell tumor, metacarpal reconstruction, osteoarticular graft, hand surgery, donor site management

## Abstract

A 36-year-old female with recurrent giant cell tumor (GCT) of the left fifth metacarpal was treated with *en bloc* resection and free osteoarticular metatarsal graft from the fourth metatarsal. Donor site management involved primary closure without bone reconstruction. The patient experienced excellent functional outcomes at 2-year follow-up, with a stable metacarpophalangeal joint and an active range of motion of 0–80 deg. No donor site complications were reported, and radiographs demonstrated successful graft incorporation and preserved joint space. The simplified donor site management reduced surgical complexity without compromising outcomes. Free osteoarticular metatarsal graft of the fourth metatarsal offers a function-preserving option for managing recurrent GCT of the hand, with minimal morbidity and durable results.

## Introduction

Giant cell tumor (GCT) of bone is a rare but aggressive benign tumor, comprising ~5% of primary bone tumors [1]*.* It typically affects long bones such as the distal femur, proximal tibia, and distal radius. GCTs in the hand, however, are uncommon, accounting for only 2%–5% of cases [1]. These tumors often present at an advanced stage in the hand, resulting in extensive bony destruction, increased recurrence rates, and more complex management [2]. Among hand bones, metacarpals are less commonly involved compared with phalanges [3, 4].

The treatment of GCTs in the hand varies, ranging from curettage (with or without bone grafting) to *en bloc* resection or ray amputation [4]. Simple curettage, while less invasive, is associated with high recurrence rates, reaching up to 50%, particularly in aggressive forms of the disease [2]. *En bloc* resection is favored for recurrent cases, often followed by reconstructive procedures to preserve hand function. Free osteoarticular transfer, also known as non-vascularized osteoarticular transfer, particularly from the metatarsals, offers a reconstructive approach that can preserve joint movement and minimize morbidity [5]. Despite its potential, there are limited reports of this technique being used for metacarpal GCTs, making it a relatively novel solution.

This case report describes a 36-year-old female with recurrent GCT of the fifth metacarpal, treated with free osteoarticular transfer of the fourth metatarsal, with primary closure of the donor site without graft reconstruction—an unconventional approach. Written informed consent was obtained from the patient for publication of this case report and accompanying images.

## Case presentation

A 36-year-old female presented with a 6-month history of swelling and pain on the dorsum of her left hand, specifically at the fifth metacarpal region. The patient experienced mild discomfort during gripping activities. Ten years prior, she had undergone treatment for GCT at the same site with curettage and local adjuvant therapy.

**Figure 1:**
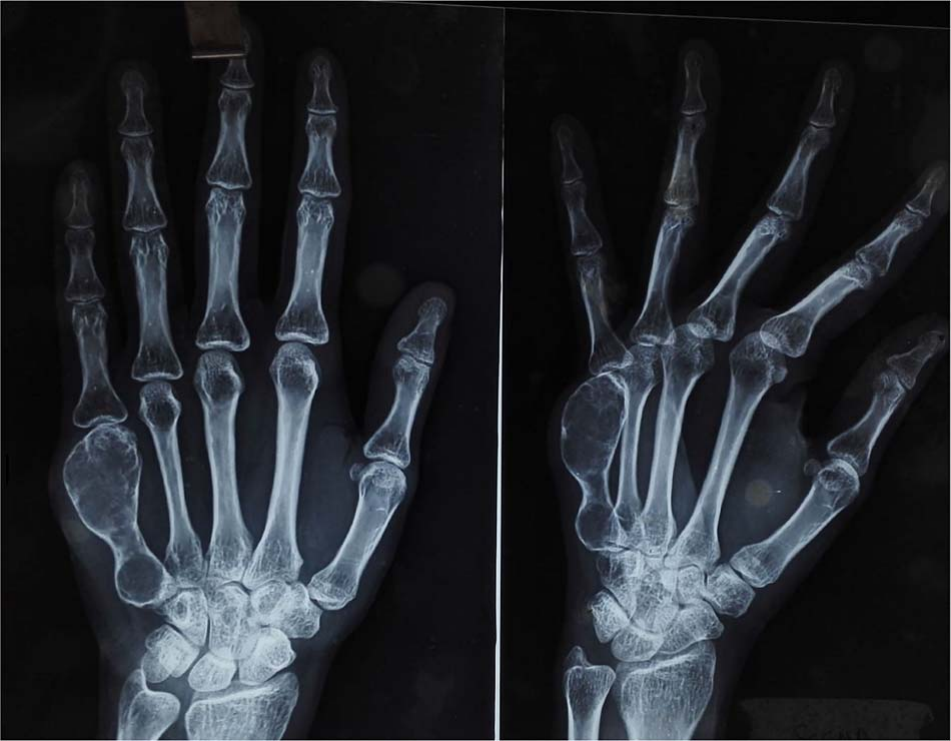
X-ray of the right hand shows an expansile, lytic lesion in the diaphysis of the fifth metacarpal with a “paper-thin” cortex extending to the subchondral bone.

On physical examination, there was a firm, fusiform swelling over the fifth metacarpal without skin changes or tenderness. The range of motion in adjacent joints was preserved. Radiographs revealed a large, expansile lytic lesion in the diaphysis of the fifth metacarpal, with cortical thinning-indicative of recurrent GCT (Fig. 1). A biopsy confirmed the diagnosis.

### Surgical plan

Given the recurrence and extent of the lesion, *en bloc* resection of the fifth metacarpal was planned, followed by free osteoarticular transfer of the fourth metatarsal from the patient’s left foot. The surgical plan included primary closure of the donor site leaving the donor site unreconstructed was planned to reduce surgical complexity.

### Surgical technique

**Figure 2:**
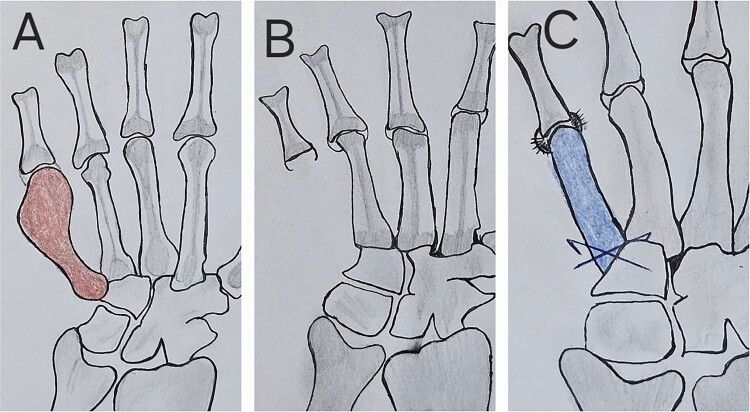
Scheme of the tumor resection and reconstruction technique with metatarsus osteoarticular graft; (A) the red shaded metacarpal indicates tumor; (B) hand after tumor resection, in which the capsule and ligaments at the MCP joint are seen; (C) free metatarsus with its capsular insertions sutured with capsule and ligaments at recipient site with k-wire fixation proximally.

(1) *En bloc resection:* Through a dorsal incision, the fifth metacarpal was exposed, and en bloc resection was performed. The resection extended to the carpometacarpal joint, preserving the metacarpophalangeal (MCP) joint capsule to maintain joint function (Fig. 2).
(2) *Harvesting the fourth metatarsal:* The fourth metatarsal was harvested using a dorsal approach to the left foot. The procedure involved preserving capsuloligamentous structures to facilitate joint reconstruction at the MCP joint of the hand. Measurements of the resected metacarpal (5.2 cm) were taken, and the donor metatarsal was trimmed to match these dimensions.
(3) *Reconstruction:* The harvested fourth metatarsal was transferred to the left hand. It was fixed proximally to the hamate using two 2-mm K-wires. The capsuloligamentous structures of the donor metatarsal were sutured to the recipient MCP joint capsule to ensure stability. The skin was closed, and the hand was immobilized with a below-elbow volar slab.
(4) *Donor site management:* The donor site was closed without bone grafting or reconstruction. The adjacent metatarsals provided adequate structural support. The patient was instructed on protective weight-bearing on below elbow plaster slab to prevent complications during the early postoperative period.

### Postoperative management and follow-up

The patient had an uneventful recovery. Hand physiotherapy was initiated 4 weeks after surgery, with progressive passive motion followed by active mobilization. Foot mobilization was encouraged once the donor site had healed sufficiently. Healing was assessed based on the absence of pain, swelling, and intact skin coverage.

**Figure 3:**
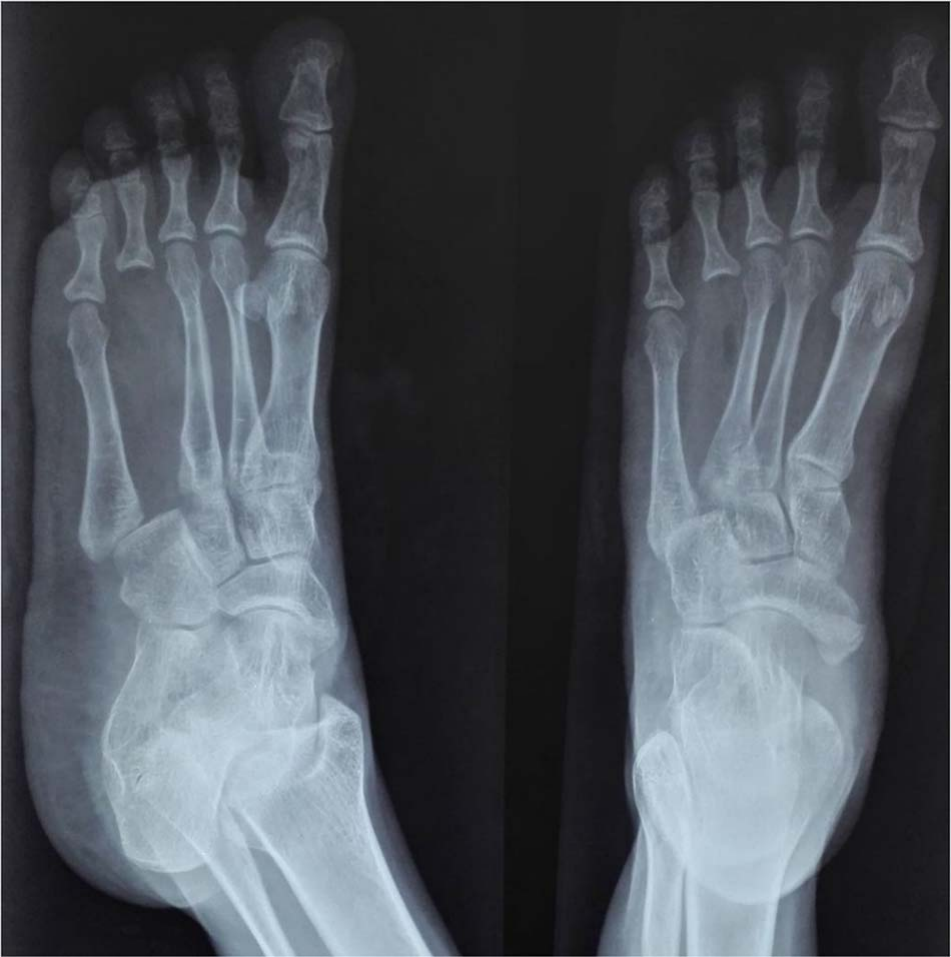
Post-operative radiograph showing preserved joint space and structure of transferred metatarsal.

**Figure 4:**
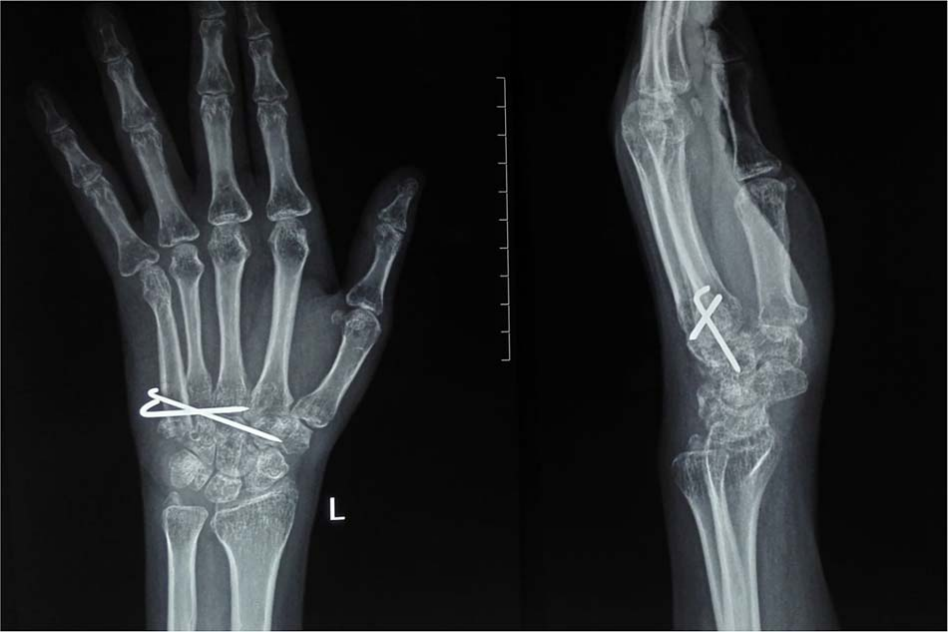
Donor site from where fourth metatarsus was harvested.

**Figure 5:**
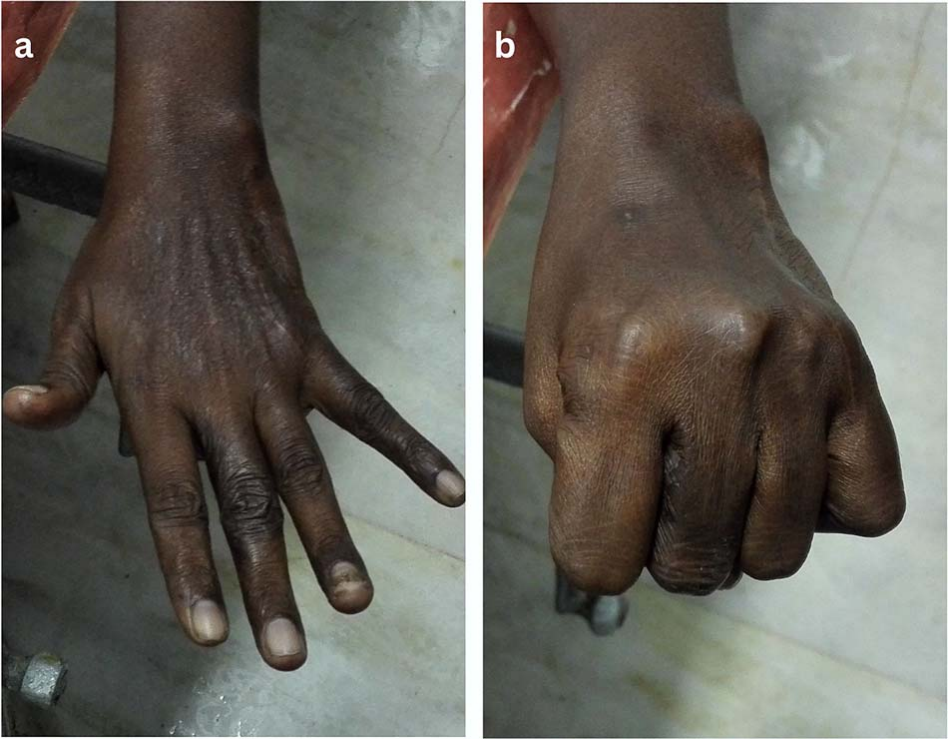
Hand function after 2 years (a; extension, b; flexion).

At 2 years postoperatively, the patient showed excellent functional outcomes. Radiographs demonstrated a stable MCP joint with well-preserved joint space, indicating successful graft incorporation (Fig. 3). Donor site radiographs at 2 years showed appropriate spacing between the third and fifth metatarsals with no evidence of arch collapse or stress reaction in the adjacent bones (Fig. 4). The patient achieved an active MCP joint range of motion of 0–80 deg (Fig. 5).

## Discussion

The surgical management of GCTs in the metacarpals is challenging, requiring both oncological control and function preservation. *En bloc* resection, although more aggressive than curettage, offers superior local control, especially in recurrent cases. Studies indicate that recurrence rates drop significantly with *en bloc* resection compared with curettage, which can have recurrence rates as high as 50% [6, 7].

Free osteoarticular transfer of the metatarsal is a reliable option for reconstructing the excised metacarpal, allowing for function preservation. This approach follows established principles of joint reconstruction and aligns with literature reporting good functional outcomes [8–10]. The omission of donor site reconstruction in this case represents a departure from traditional methods, potentially simplifying the procedure and reducing surgical time and morbidity [11, 12].

The functional outcomes in this case are comparable with other reported cases. The patient achieved a satisfactory range of motion, grip strength, and ability to perform complex hand functions. The minor restriction in flexion observed is a known limitation of the technique, typically discussed with patients before surgery.

This technique offers several advantages over other reconstructive options, including its relative technical simplicity and feasibility for general orthopedic surgeons, without requiring microsurgical expertise. Critical steps for success include careful preservation of capsuloligamentous structures, precise length matching, and secure fixation.

Long-term surveillance showed stable graft incorporation, preserved joint space, and maintained density of the transferred metatarsal, supporting the biological effectiveness of the procedure. However, continued follow-up is necessary due to the potential for late recurrence in GCT cases.

## Conclusion

This case demonstrates that free osteoarticular transfer of the fourth metatarsal is a viable and function-preserving option for treating recurrent GCT of the metacarpal. It provides excellent outcomes with minimal donor site morbidity. Selective omission of donor site reconstruction can be considered in carefully selected cases, potentially simplifying the surgical procedure without compromising results.
